# The Genetic Spectrum of Familial Hypercholesterolemia (FH) in the Iranian Population

**DOI:** 10.1038/s41598-017-17181-9

**Published:** 2017-12-06

**Authors:** R. H. Fairoozy, M. Futema, R. Vakili, M. R. Abbaszadegan, S. Hosseini, M. Aminzadeh, H. Zaeri, M. Mobini, S. E. Humphries, A. Sahebkar

**Affiliations:** 1 Cardiovascular Genetics, Institute of Cardiovascular Science, University College London, London, United Kingdom; 2 Molecular Diagnostic Unit, Clinical Laboratory Department, King Abdullah Medical city in Makkah, Makkah, Saudi Arabia; 3 Centre for Cardiology in the Young, Institute of Cardiovascular Science, University College London, London, United Kingdom; 4 Department of Pediatric Endocrinology and Metabolism, Faculty of Medicine, Mashhad University of Medical Sciences, Mashhad, Iran; 5 Medical Genetics Research Center, Mashhad University of Medical Sciences, Mashhad, Iran; 6 Diabetes Research Center, Ahvaz Jundishapur University of Medical Sciences, Ahvaz, Iran; 7 Neonatal and Children Health Research Centre, Golestan University of Medical Sciences, Gorgan, Iran; 8 School of Medicine, Mashhad University of Medical Sciences, Mashhad, Iran; 9 Biotechnology Research Center, Pharmaceutical Technology Institute, Mashhad University of Medical Sciences, Mashhad, Iran; 10 School of Pharmacy, Mashhad University of Medical Sciences, Mashhad, Iran

## Abstract

Familial hypercholesterolemia (FH) is an autosomal dominant disorder associated with premature cardiovascular disease (CVD). Mutations in the *LDLR*, *APOB*, and *PCSK9* genes are known to cause FH. In this study, we analysed the genetic spectrum of the disease in subjects from the Iranian population with a clinical diagnosis of FH. Samples were collected from 16 children and family members from five different cities of Iran. Probands were screened for mutations in the *LDLR*, *APOB*, and *PCSK9* genes using next generation sequencing, with results confirmed by Sanger sequencing. The likely pathology of identified variants was examined using *in silico* tools. Of the probands, 14 had a clinical diagnosis of homozygous FH and two of heterozygous FH. No mutations were found in either *APOB* or *PCSK9*, but nine probands were homozygous for seven different *LDLR* mutations, with p.(Trp577Arg) occurring in three and p.Val806Glyfs*11 occurring in two patients. Two mutations were novel: p.(Leu479Gln) and p.(Glu668*). Seven probands with a clinical diagnosis of FH were mutation negative. This pilot study, integrating clinical and molecular-based techniques, begins to elucidate the FH heterogeneity and the mutation spectrum in the Iranian population. Such information is important for future disease management and cost savings.

## Introduction

Familial hypercholesterolemia (FH) is an autosomal dominant disorder resulting in elevated plasma low-density lipoprotein cholesterol (LDL-C). Heterozygous FH (HeFH) is common, with an estimated prevalence of at least 1/500^1^ in most European populations, but being higher in some populations such as the Danes^2^, the Afrikaners in South Africa^3^, French Canadians^4^ and the Dutch^5^, with recent estimates in the UK suggesting around 1/270^6,7^. Plasma LDL-C in HeFH is two-to-three-fold higher than normal. Individuals with this form of FH are more likely to develop premature coronary heart disease (CHD) in the second or third decade of their lives^1^. The second form is homozygous FH (HoFH), which has an estimated prevalence of 1 in 1 million in most populations, however, some populations have reported higher prevalence such as 1:300,000 in the Netherlands^5^. Individuals with HoFH have six-to-eight-fold higher levels of LDL-C plasma than normal and develop CHD in the early stages of their lives, often dying before the age of 20^1^. FH is caused by a mutation in one of three genes: the low-density lipoprotein cholesterol receptor (*LDLR)*, Apolipoprotein B gene (*APOB)*, or a gain-of-function mutation in the gene for proprotein convertase subtilisin/kexin type-9 (*PCSK9*). Autosomal recessive hypercholesterolemia (ARH), a rare inheritance of FH, has been reported^8^ and occurs when individual inherits two pathogenic variants in the low-density lipoprotein adaptor protein 1 (*LDLAP1*) gene. In the UK, the vast majority (93%) of identified mutations are found in the *LDLR* gene, a further 5% in *APOB*, and about 2% in the *PCSK9* gene^9,10^.

FH can be diagnosed using well-established clinical criteria. The Simon Broome Register Criteria (UK)^11^ and The Dutch Lipid Clinic Network (DLCNC)^12^ (Europe) use total cholesterol (TC) and LDL-C levels, presence of tendon xanthoma (TX), a family history of hypercholesterolemia and premature CHD in a first and/or second degree relative. The most common cholesterol-lowering drugs are statins, which are used as the primary intervention method to prevent premature CHD. However, statin efficacy ranges from a 30% to 60% reduction in LDL-C, which depends on statin potency, type and dose, and mutation type^13^. Identifying and treating FH patients in childhood or early adulthood is important to prevent or reduce morbidity and mortality from premature CHD^14^.

Although the FH molecular basis and prevalence have been demonstrated in many populations, the FH prevalence and spectrum are unknown in Iran. In 2015, clinical practice guidelines for the diagnosis and treatment of HoFH in the Middle East region was published, which showed several factors such as consanguineous marriages, treatment accessibility and cascade screening limitations that were not found in Western countries^15^. Previous Iranian FH Genetic studies have used limited genetic screening methods based on PCR technology^16–20^. Fard-Esfahani *et al*. in 2005, who examined 30 patients with clinical possible FH, identified one novel mutation in exon 4 of the LDLR p.(Gly445Cy) using the PCR-single-strand conformation polymorphism (PCR-SSCP) method^18^, while another study used PCR-Restriction Fragment Length Polymorphism (PCR-RFLP) and PCR-SSCP methods, where no mutations were reported either in the promoter or coding region of *LDLR* or in exon 26 of *APOB* ^16,17^. A recent study examined 80 patients with a clinical diagnosis of FH, and after testing for the two common *APOB* mutations and screened the *LDLR* gene only for exons 3, 4, 9 and 10 only, identified two mutations in exons 3 and 4^20^. We believe that our study, using Next Generation Sequencing for the whole of *LDLR/APOB/PCSK9*, is the first to carry out a thorough genetic screen in Iranian FH patients.”

HoFH may have a higher prevalence in Iran because consanguineous marriages are more common in the Persian culture (35–54%)^21^. Historically, a patient was clinically diagnosed with HoFH, if he/she had untreated LDL-C > 13 mmol/L (500 mg/dL) or treated LDL-C > 8 mmol/L (300 mg/dL), and the presence of TXs before the age of 10 years, or the patient’s parents were diagnosed with HeFH^22^. However, the range of LDL-C levels reported in HoFH patients is broad and can overlap with ranges found in other types of FH^15,22^, which is another challenge for identification. Two cases of Iranian children with HoFH have reported severe septum and complex CHD outcomes^23,24^. Therefore, it has become essential to identify FH patients in Iran, and find diagnostic strategies and appropriate treatment in order to reduce morbidity and mortality due to CHD. This study provides a diagnostic guideline of FH in Iran including clinical criteria, cascade screening by using the next generation sequencing (NGS)-based method followed by Sanger sequencing, in addition to mutation pathogenesis analyses.

## Results

### Patient characteristics and sample selection

Clinical and lipid parameter details of the 57 recruited participants are shown in Supplementary Table S2. We selected 16 probands from unrelated families. Fourteen probands had been diagnosed with clinical HoFH, had LDL-C > 13 mmol/L or treated LDL-C > 8 mmol/L, TX, and a positive family history of hyperlipidaemia or MI. Two probands (FH9-P1 and FH10-P2) were suspected of having FH, and as shown in Supplementary Table 2, although their treated cholesterol levels did not exceed the FH cut-off (LDL-C = 3.3 and 4.9 mmol/l, respectively), but they reported a strong family history of hyperlipidaemia and/or MI. The baseline characteristics of the16 selected cases are shown in Table 1.

**Table 1:** Baseline characteristics (mean ± SD) used in this study.

Variable	Probands
Sample size	16
Age (in years)	7.1 (±4.8)
Total Cholesterol (mmol/l)	16.3 (±5.7)
LDL-Cholesterol (mmol/l)	12.0 (±4.6)
Tendon xanthomata	14 (87.5%)
Family history of CHD	6.0 (37.5%)
Family history of hyperlipidemia	16 (100%)
Taking lipid-lowering medication	16 (100%)

### Mutation Spectrum

Out of the 16 patients examined, a FH-causing variant was found in nine patients (56%), while no mutation was detected in seven individuals (44%), five clinically diagnosed with FH and two suspected of FH. All identified mutations were found in *LDLR*. As shown in Table 2, although TC and LDL-C levels were higher in the mutation positive group compared to the mutation negative group, in this small sample these differences were not statistically significant (TC p = 0.10 and LDL-C p = 0.09).

**Table 2:** Familial hypercholesterolemia (FH) patient characteristics: mutation positive and negative groups in the Iran study.

Variable	Mutation positive	Mutation negative	p-value
Sample size	9	7	
Age (in years)	7.8 (±5.6)	6.3 (±3.8)	0.56
Total Cholesterol (mmol/l)	18.4 (±4.6)	13.7 (±6.7)	0.10
LDL-Cholesterol (mmol/l)	13.6 (±3.8)	9.6 (±5.0)	0.09
Tendon xanthomata	9 (100%)	5 (71.4%)	0.16
Family history of CHD	5 (55.5%)	1 (14.3%)	0.12
Family history of hyperlipidemia	9 (100%)	7 (100%)	—
Taking lipid-lowering medication	9 (100%)	7 (100%)	—

Mean and Standard deviation (SD), where appropriate, are shown. P-value was determined using ANOVA for numeric variables and chi-squared test for categorical variables.

**Figure 1:**
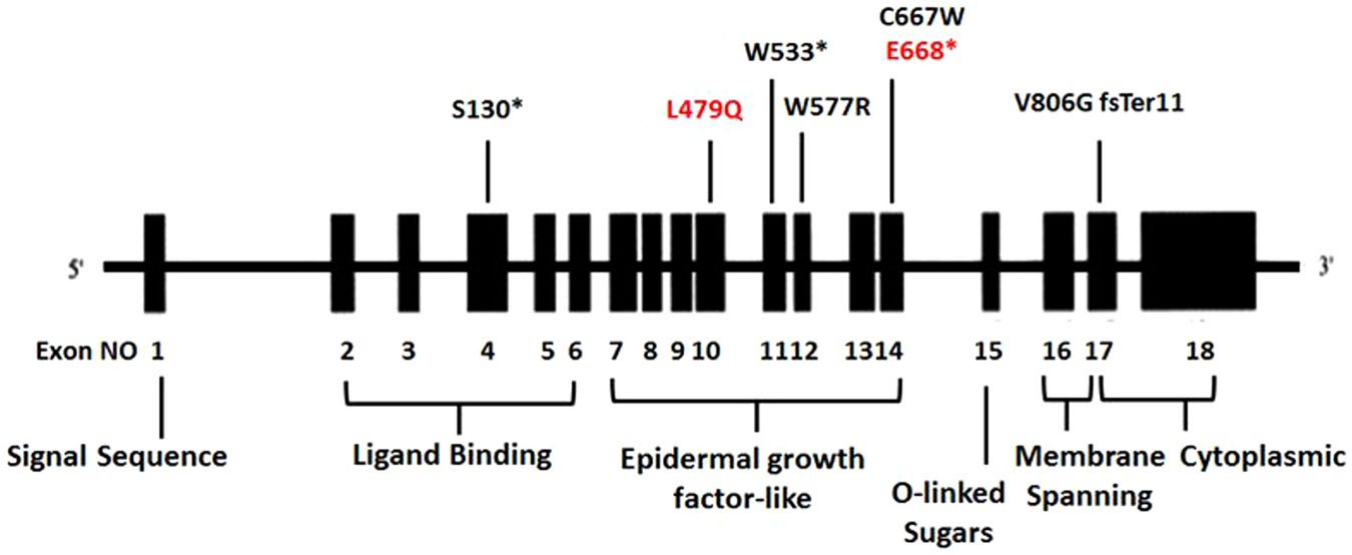
Diagram of *LDLR* gene showing the mutations identified in this study. Seven mutations were identified in this study: two novel mutations are indicated in red. Exons are shown as vertical boxes and introns as the lines connecting them.

In eight patients with a clinical diagnosis of HoFH, we identified six different *LDLR* mutations (Table 3, Fig. 1). The DNA mutations were reported in five different exons of *LDLR* (4, 10, 11, 12, and17). Five of these identified mutations have been reported in the UCL-FH mutation database. Three nonsense mutations were identified, c.389 C > G, p. (Ser130*) in exon 4, and c.1599 G > A, p.(Trp533*) in exon 11 and p.(Cys668*). Also, we found missense mutation, c.1729T > C p.(Trp577Arg) in exon 12. Three patients carried the *LDLR* mutation p.(Trp577Arg), and one carried p.(Cys667Trp). The fifth mutation was a frame-shift mutation in exon 17, c.2146dupG p.(Val806Glyfs*11), where an insertion of G changed the amino acid at position 806 from Valine to Glycine and led to a shift in the reading frame and the creation of a stop codon downstream.

**Table 3:** Identified *LDLR* mutation in an Iranian patient with FH.

ID	TC mmol/l	LDL-C mmol/l	Exon	Base pair change	Amino acid change	PolyPhen	SIFT	Mutation Taster	Reported FH causing	Country report
FH15-P	18.6	13.0	Exon 4	c.389 C > G	p.(Ser130*)	N/A	N/A	N/A	55	Denmark
FH14-P	16.5	15.0	Exon 10	c.1436 T > A	p.(Leu479Gln)	D	D	D	Novel	Iran
FH8-P	21.7	19.4	Exon 11	c.1599 G > A	p.(Trp533*)	D	D	D	56	Subject originality unknown
FH1-P	11.4	10.0	Exon 12	c.1729T > C	p.(Trp577Arg)	D	D	D	25,26	Turkey
FH2-P	18.6	14.2								
FH7-P	22.5	17.5								
FH17-P	15.3	13.6	Exon 14	c.2001_2002delinsGT	p.(Cys667Trp)	D	D	D	57	France
					p.(Glu668*)	N/A	N/A	N/A	Novel	Iran
FH3-P	21.5	16.1	Exon 17	c.2146dupG	p.(Val806Glyfs*11)	N/A	N/A	N/A	28,29,30,31 and 32	US, Sweden, Czech, Nether-lands, Japan
FH5-P	15.5	7.8								

D: Deleterious, P: Possibly damaging, B: Benign, N/A: not applicable.

Three patients (FH 4-P, FH 12-P, and FH 16-P) were clinically diagnosed with HoFH, however, no mutation was found in any of the FH genes. Also, no mutation was found in the subjects with suspected FH.

### Novel *LDLR* variants

**Figure 2:**
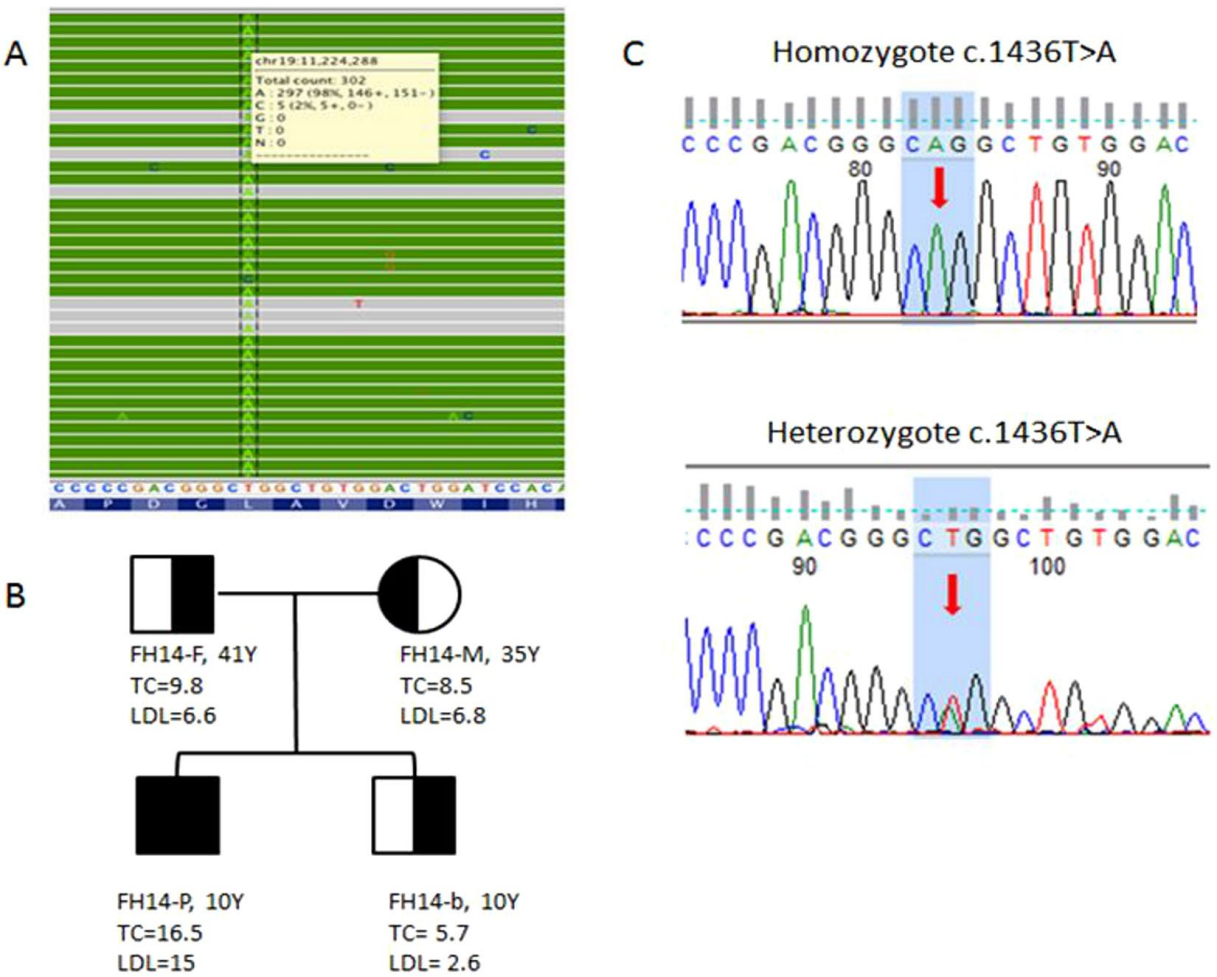
Co-segregation analysis of family with *LDLR* novel mutation p(.Leu479Gln). (**A**) NGS sequencing reads alignment using Integrated Genomics Viewer (http://software.broadinstitute.org/software/igv/) for proband FH14-P reports a variant at exon 10 of the LDLR gene [c.1436 T > A, p(.Leu479Gln)], the calling rate A allele of the variant is 95% which indicates the proband is a homozygote for the variant. (**B**) Co-segregation analysis of the FH14 family shows the 3 other family members are heterozygotes of the variant. (**C**) Sanger sequencing shows a base change between homozygote and heterozygote inherited modes.

The functionality of the missense mutations was assessed by *in-silico* mutation prediction tools, including PolyPhen2, SIFT, and Mutation Taster, and were predicted to be functional. As the nonsense mutations and insertion frame-shift mutation led to peptide truncation, they were also predicted to be functional. We found two novel mutations in *LDLR* in two HoFH probands, which are not reported in the UCL-FH mutation database, one in exon 10 c.1436 T > A, p.(Leu479Gln) and another in exon 14 c.2001_2002delinsGT p.(Glu668*). The mutation heritability of the mutations was confirmed by investigating the proband’s family members. The homozygous p.(Leu479Gln) variant was found in a 10year-old boy who had high levels of TC (16.5 mmol/l) and LDL-C (15 mmol/l). Co-segregation analyses showed that this mutation is an FH-causing mutation and was present in all members of the family including both parents and a sibling (Fig. 2). They were all heterozygous for this mutation; both parents had raised cholesterol levels [Father (FH14-F): TC = 9.8 mmol/l, LDL-C = 6.6 mmol/l; Mother (FH14-M): TC = 8.5 mmol/l, LDL-C = 6.8 mmol/l], but not the sibling.

**Figure 3:**
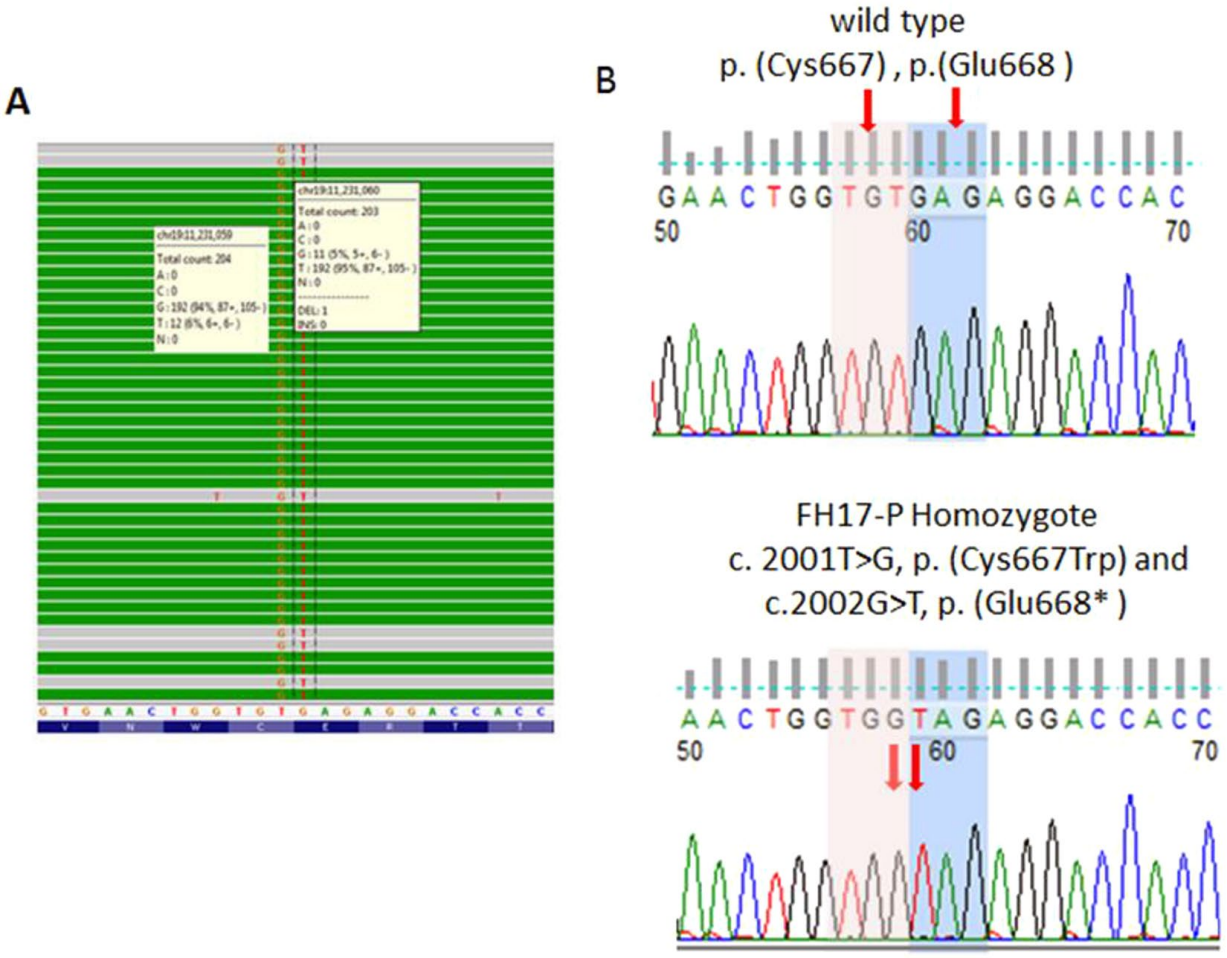
*LDLR* novel mutation p.(Glu668*) sequencing data. (**A**) NGS sequencing reads alignment using Integrated Genomics Viewer (http://software.broadinstitute.org/software/igv/) for proband FH17-P reports two base change c.2001_2002delinsGT at exon 14 of the LDLR gene which affecting two amino acid [p.(Cys667Trp) and p.(Glu668*)], the calling rate of minor alleles of the variants are ≥94% which indicate the proband is a homozygote of the variants. (**B**) Sanger sequencing shows a base change between wild type and mutant homozygote inherited mode.

The second novel mutation, p.(Glu668*),was found in a 4 year-old girl who had high levels of TC (15.0 mmol/l) and LDL-C (13.6 mmol/l), but no family history of either hyperlipidaemia or MI. It was also found that the proband was homozygous for another mutation that changes amino acid 667, p.(Cys667Trp) (Fig. 3), but due to the presence of the sequence change creating the nonsense mutation at position 668, we designate the pathogenic mutation in this subject as p.(Glu668*). Unfortunately, family members of the proband were not available for segregation analysis.

## Discussion

This study has identified a number of previously reported as well as novel mutations among patients with a clinical diagnosis of HoFH and HeFH attending various Iranian clinics. Amongst the 14 child probands clinically diagnosed with HoFH, mutations were found in nine patients (57%). No mutations were found in two additional child patients included because of having a strong family history of hyperlipidaemia and/or MI. Different mutations were identified using the targeted NGS method and were confirmed by Sanger sequencing, all in *LDLR*, where five had been previously reported as FH-causing mutations, but were not previously reported in the Iranian population^20^, and two, p.(Leu479Gln) and p.(Glu668*) that were first reported in this study.

This study has found two mutations occurring in more than one of the patients examined. The mutation p.(Trp577Arg), which was seen in three patients with HoFH. This was previously reported as very common in the Turkish population, explaining 21.4% of the predicted defective *LDLR* alleles^25,26^. For populations that are geographically close, genetic and geographic distances are often highly correlated^27^. Since there is close geographical and cultural links between Turkey and Iran it it very likely that they will share some common founder mutations. The three probands were from Esfarayen (FH1-P, FH2-P) and Guchan (FH7-P) which are in the north-east of Iran on the border with Turkmenistan, and 74% of the Turkmenistan population are Turkmen (Turkish background).

The second mutation found in more than one patient in the study was a frame-shift mutation p.(Val806Glyfs*11) in exon 17, which was found in two patients with HoFH. This mutation has been reported in different populations in the USA^28^, South Sweden^29^, Czech^30^, Netherlands^31^ and Japan^32^. There are two possible explanations for the presence of the same mutation in different populations. First, there may be an ancestral mutation occurring in an individual living somewhere in the European continent, which has then spread throughout the world due to human migration. The FDB mutation p.(R3527Q), for example, is an ancestral mutation found in western Europe 6000–7000 years ago, and distributed to the East and West via human migration in the past 2000–3000 years^33^. This possibility could be tested by determining if the mutation occurs on the same SNP haplotype, but data is not available to carry this out.

The second possible explanation is that the mutation may be located in a CpG (5′—C—phosphate—G—3′) mutation hotspot^34^. Cytosine in the CpG dinucleotide carries a methyl group which is added via methyltransferase to form 5-methylcytosine, which causes DNA methylation and affects gene transcription via preventing transcription factors and other proteins from binding^35^. However, by looking for the sequence around the point mutation and the CpG island map of *LDLR* gene, the insertion of guanine (G) nucleotide (c.2416_2417insG) does not create a CpG sequence (Figure S1). As shown in the *LDLR* CpG island map, exon 17 was not included in any CpG island (Figure S2). The literature reported 12 CpGs in the *LDLR* promoter in atherosclerosis patients^36^. However, it can be seen by inspection of the gene sequence at this point that the mutation is due to the duplication of a G in a homonucleotide tract of 5 Gs, which is frequently a mutational cause. At the present time it is not possible to distinguish between these two possibilities.

Four of the identified mutations in this study have been reported in the UCL-FH mutation database, and are predicted to be pathogenic. Two identified mutations are nonsense mutations: p.(Ser130*) (exon 4) is located in repeat three of the ligand binding domain of the receptor and p.(Trp533*) (exon 11) encodes the EGF-like domain. Both mutations create a premature stop codon resulting in peptide truncation and non-functional protein products. Also one missense mutation p.(Trp577Arg) was identified in the region of the gene that encodes the EGF-like domain. Variants in this domain block receptor dissociation in the endosome and this influences the machinery responsible for recycling receptors to the cell surface, which causes the disease phenotype. The exon 12 mutation p.(Trp577Arg) is located in the third Y-repeat (five repeats containing a YWTD motif) of the EGF-like domain of the LDL-R, where other missense mutations have been functionally characterized to be class 2 mutations (transport-defective alleles)^1,37^. Thus, we propose that the p.(Trp577Arg) mutation reported here would cause the same class of deficiency due to its location. The fourth mutation was identified in Exon 17 p.(Val806Glyfs*11) which encodes membrane-anchoring and cytoplasmic tail domains which are vital for locating the receptor correctly in the cell membrane, thus the truncated peptide at this position affects this machinery and leads to the disease phenotype.

Two novel mutations have been identified in *LDLR* that were not reported in the UCL-FH mutation database: a missense mutation in exon 10 p.(Leu479Gln) and a truncated peptide in exon 14 p.(Glu668*). Both mutations are encoded in the EGF-like domain of *LDLR*, which is known to be important for receptor dissociation in endocytosis and receptor recycling to the cell surface, and are thus pathogenic. Analysis of the proband family members carrying p.(Leu479Gln) showed that the mutation is heritable and causing FH.

Forty-three percent of subjects examined in this study were FH-mutation negative. It has been demonstrated that the elevated LDL-C levels in mutation negative subjects with a clinical diagnosis of FH are due to accumulation of LDL-C raising variants “polygenic FH”^38^. There are 12 common “polygenic” LDL-C raising variants described in the European population (MAF > 0.05%), however, the frequency of these variants in the Iranian population has not yet been studied, However, based on data from other countries, it is likely that the elevated levels of LDL-C seen in at least some of the patients who are FH- mutation negative have a polygenic and not a monogenic explanation, and this may be a possible explanation for the high cholesterol phenotype in the two non HoFH additional child probands FH9-P1 and FH10-P2. Studies have also showed mutations in *ABCG5/8* may cause the phenotype of very elevated cholesterol levels^39^, and these genes could be examined in the mutation-negative patients in the future. The other possible explanation for the lack of a mutation in the three known FH genes could be that the hypercholesterolaemia is due to a mutation in an undiscovered gene(s). Recently, mutations in Signal-transducing adaptor protein 1 (*STAP1*) gene have been reported in Dutch families who had the FH phenotype but were mutation-negative for the three FH genes^40^, and this gene could also be examined in the future.

Two probands (7 and 12 years) suspected of having FH were included in this study, although their cholesterol levels did not exceed the FH cut-off, but they reported a strong family history of hyperlipidemia and MI. No mutations were found in any of the three tested FH genes in these patients. In both patients the presence of TX, a family history of CHD and hyperlipidaemia led to testing with a further lipoprotein screen: high levels of Lipoprotein(a) (Lp(a)) were found in both probands (Lp(a) > 100 mg/dL but with LDL-C levels within the normal range (~3.3 mmol/l) when undergoing lipid-lowering drug therapy). An elevated plasma concentration of Lp(a) has been reported as an independent risk factor for CVD^41–43^. The clinical phenotype of Lp(a) hyperlipidemia (Lp(a)-HLP) shows great variability, as does FH, however the diagnosis of Lp(a)-HLP can be determined by the plasma concentration of Lp(a)^44^.

### Limitation

There were some limitations in this study. One was the small sample size, which means we cannot extrapolate with any certainty to the overall prevalence of the detected mutations in FH patients in Iran. Also, in many countries where this has been examined, 5–10% of FH patients carry a gross insertion or deletion of the *LDLR* gene and in order to obtain a full molecular characterization of the mutation spectrum in the Iranian population screening for this type of defect should have been carried out. However at the current time an appropriate method to do this is not up and running in our laboratory, and unfortunately we do not have sufficient DNA to carry this out. The NGS method should have allowed us to look for any such gross deletions and insertions but again the quality of the DNA was inadequate to allow us to perform this with accuracy. Also it was not possible to perform segregation analysis for all patients with novel variants due to communication difficulties with their relatives living in different parts of the country. Another limitation was that polygenic FH was not examined in the mutation negative group due to lack of reports about polymorphism frequency in the Iranian population, and the gene encoding Apo(a) was not screened because of grant and time limitations.

## Conclusion

Although more than 1,700 variants have been reported in *LDLR* ^45^, there are still novel variants being found, proving the heterogeneity of FH. In this work we showed how we selected 16 FH patients as probands out of 57 based on DLCN clinical criteria, and then 14 probands were diagnosed as HOFH based on cholesterol levels. To confirm the clinical diagnoses genetic tests were performed, which indeed confirm the clinical diagnosis in 57% of the subjects. Among the seven mutations identified in this study, two variants were novel (previously unreported). Also we show that for a novel variant it is important to run family genetic analysis to confirm co-segregation of the variant and high cholesterol levels, and thus the pathogenicity of the mutation. We think our work gives a guideline for physicians for how to integrate clinical observations and genetic tests for patient benefit. It is recommended that FH-mutation negative subjects with a clinical diagnosis of FH are examined for 12 common “polygenic” LDL-C raising variants. Also, a genetic screening for the Apo (a) gene in patients with Lp(a)-HLP is recommended. Further and larger studies are needed to more fully elucidate the frequency of FH causing mutations in Iran and to identify common and unique mutations in subjects of Persian ancestry.

## Methods

### Subjects

Fifty-seven samples were collected from 16 child probands and family members from different paediatric and endocrinology clinics in five different cities (Ahvaz, Bandar Abbas, Esfarayen, Mashhad and Torbat Heydariye), which represents three regions of Iran. Clinical diagnosis was made according to the Dutch Lipid Clinic Network Criteria (DLCNC) as previously described^12^. The probands were diagnosed with clinical definite FH (DFH) if they had a total cholesterol >6.7 mmol/l or LDL-C > 4.0 mmol/l if <16 years and, TX or TX in a first or second degree relative. They were diagnosed with clinical probable FH (PFH) if they had a high cholesterol as DFH and a family history of myocardial infarction (MI) in a first degree relative <60 years or a family history of total cholesterol l >7.5 mmol/l in a first or second degree relative. Also, any child who was suspected of having FH but who did not meet the criteria still underwent analysis for *LDLR*, *APOB* and *PCSK9* mutations because if the child was from a family with PFH, there is a high probability that he/she has FH. For this reason, samples were collected from the proband and any first degree relatives.

### Blood sampling and DNA isolation

This study was performed on 16 children and their family members from five different cities of Iran. All families were diagnosed for FH according to the DLCNC criteria. Information on consanguinity was obtained by questionnaire. The study procedures were approved by the Ethics committee (approval code #IR.MUMS.fm.REC1394142) of the Mashhad University of Medical Sciences, Mashhad, Iran. All patients’ parents and/or their guardians gave their written informed consent to participate in this study.

Blood samples (3 mL) were obtained from all patients and their available parents were collected in EDTA tubes. Genomic DNA was extracted from peripheral blood samples using the standard salting-out method^46^ with the High Pure PCR Template Preparation Kit (version 20).

### Next generation sequencing (NGS)

A targeted-NGS method was used. Primers to amplify coding regions (±25 base pairs (bp)) of the three autosomal dominant FH genes (*LDLR*, *APOB*, *PCSK9*), and the autosomal recessive FH gene (*LDLRAP1*) for targeted sequencing were designed using the Illumina Design Studio^47^. Amplicon length was set at 250 bp. The library preparation was performed using the TruSeq Custom Amplicon (TSCA) v1.5 kit (Illumina, San Diego, CA) and sequencing (in both directions) was done using the Illumina MiSeq platform. All experiments were performed in accordance with relevant guidelines and regulations.

### Variant detection and analysis

The raw data acquired were aligned to human reference genome (Hg19). The criteria for the standard variant calling pipeline were: coverage ≥30×, a minimum of five reads for an altered allele, a Phred quality ≥20, and a strand bias filter. A sensitive pipeline was used to ensure that variants were not missed (a coverage ≥15×, a minimum of two reads for an altered allele, a Phred quality ≥ zero, and no strand bias filter). Variant annotation was carried out with Variant Effect Predictor (VEP) tool from Ensembl^48^ and based on the transcripts *LDLR*: ENTS00000558518, *APOB*: ENTS00000233242, *PCSK9*: ENTS00000302118, and *LDLRAP1*: ENTS00000374338. Reported variants were filtered based on their frequency and functional affects. Variants with minor allele frequency >1% according to the 1000 Genomes genotype data^49^ were considered non-pathogenic and excluded from further analysis. The remaining variants were flagged as *rare* (frequency = 0.005) or *novel* (frequency = 0). Also, variants were flagged as *functional* when they were most likely to affect a protein’s function, that is: non-synonymous, stop gain, stop loss, frame-shift deletions and insertions, and splice site variants. *In-silico* mutation prediction tools were also used to predict the possible impact of an amino acid substitutions on the structure and function of a human protein, including Sorting Intolerant Form Tolerant (SIFT)^50^, Polyphen-2 V2^51^, and MutationTaster^52^. Also, the variants were described as causing FH if they were reported on the UCL-FH mutation database (http://www.ucl.ac.uk/ldlr/Current/)^45,53^, which is supported by evidence in the literature. While variants were described as possible FH-causing if they were unreported in the database but they were predicted to be pathogenic *in-silico* and highly conserved (the UCSC genome browser used to analyze the conservation level of the variant position). The variants that passed filtering pipelines were directly Sanger sequenced for validation, as described below.

### Sanger sequencing

Variants were confirmed by Sanger sequencing. Oligonucleotide primers for PCR were designed to cover, where appropriate, intron–exon junctions and up to 60 bp of the introns^54^. Primers were designed to amplify the promoter and *LDLR* exons; a part of exon 26 of *APOB* that includes the p.(R3527Q) mutation and p.(D374Y) (exon 7) of the *PCSK9* gene were included for screening (Table S1). PCR was carried out in the Rotor-Gene6000 (Qiagen Ltd, Crawley, West Sussex, UK). The PCR reaction mix comprised 1.5 ng of DNA, 0.05 µl from each primer, and 5 µl AccuMelt HRM SuperMix (2x) (Quanta Biosciences) to a total volume 10 µl. The PCR conditions were as follows; started at 95 °C for 5 minutes, followed by 40–45 cycles of denaturing at 95 °C for 5 seconds, annealing at 60 °C for 10 seconds, and extending at 70 °C for 20 seconds. The amplified fragment was sequenced using Sanger sequencing. The DNA sequence was assessed manually.

## Electronic supplementary material

Supplementary information
