# Supplementary material for: The Genetic Spectrum of Familial Hypercholesterolemia (FH) in the Iranian Population
Source: Sci Rep. 2017 Dec 6;7:17087. doi: 10.1038/s41598-017-17181-9 (PMC5719081; doi:10.1038/s41598-017-17181-9)
Supplement: Supplementary file 1 — Supplementary information [file 41598_2017_17181_MOESM1_ESM.pdf]

The Genetic Spectrum of Familial Hypercholesterolemia (FH) in the Iranian Population

Fairoozy, R. H.[1, 2], Futema, M.[3], Vakili, R. [4, 5], Abbaszadegan, M. R. [5], Hosseini, S. [5] , Aminzadeh, M. [6] , Zaeri, H. [7] , Mobini, M. [8] ,Humphries, S. E.[1*], Sahebkar, A.[9]

[1] Cardiovascular Genetics, Institute of Cardiovascular Science, University College London, London, United Kingdom.

[2] Molecular Diagnostic Unit, Clinical Laboratory Department, King Abdullah Medical city in Makkah, Makkah, Saudi Arabia

[3] Centre for Cardiology in the Young, Institute of Cardiovascular Science, University College London, United Kingdom

[4] Department of Pediatric Endocrinology and Metabolism, Faculty of Medicine, Mashhad University of Medical Sciences, Mashhad, Iran.

[5] Medical Genetics Research Center, Mashhad University of Medical Sciences, Mashhad, Iran

[6] Diabetes Research Center, Ahvaz Jundishapur University of Medical Sciences, Ahvaz, Iran

[7] Neonatal and Children Health Research Centre, Golestan University of Medical Sciences, Gorgan, Iran

[8] School of Medicine, Mashhad University of Medical Sciences, Mashhad, Iran

[9] Biotechnology Research Center, Mashhad University of Medical Sciences, Mashhad, Iran

***Corresponding author:** Professor Steve E Humphries, Centre for Cardiovascular Genetics, Institute of Cardiovascular Science, 5 University Street, University College London, London, UK WC1E 6JF, Tel 0207 679 6962 , email: steve.humphries@ucl.ac.uk

Supplementary

Figures

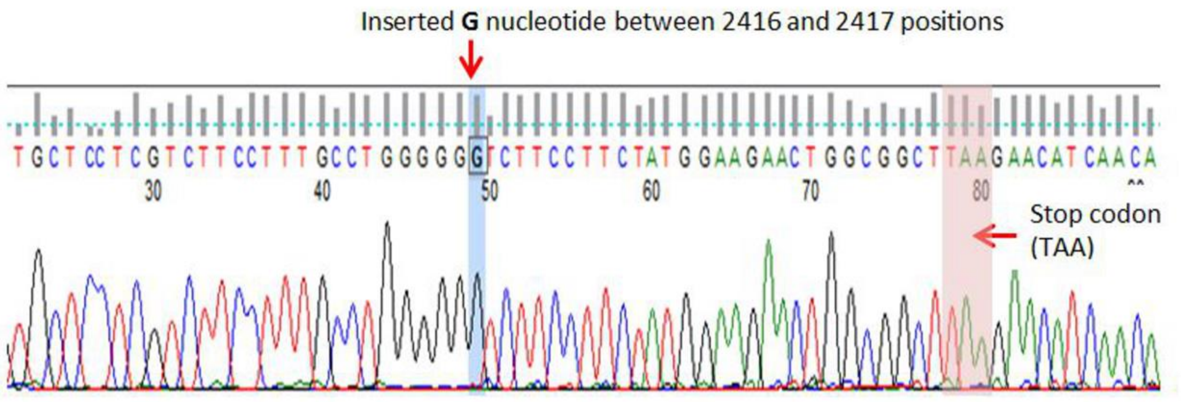

Figure S1: Electropherogram for DNA sequence analysis of 19: c.2416_2417insG (LDLR, p.Val806Glyfs*11)

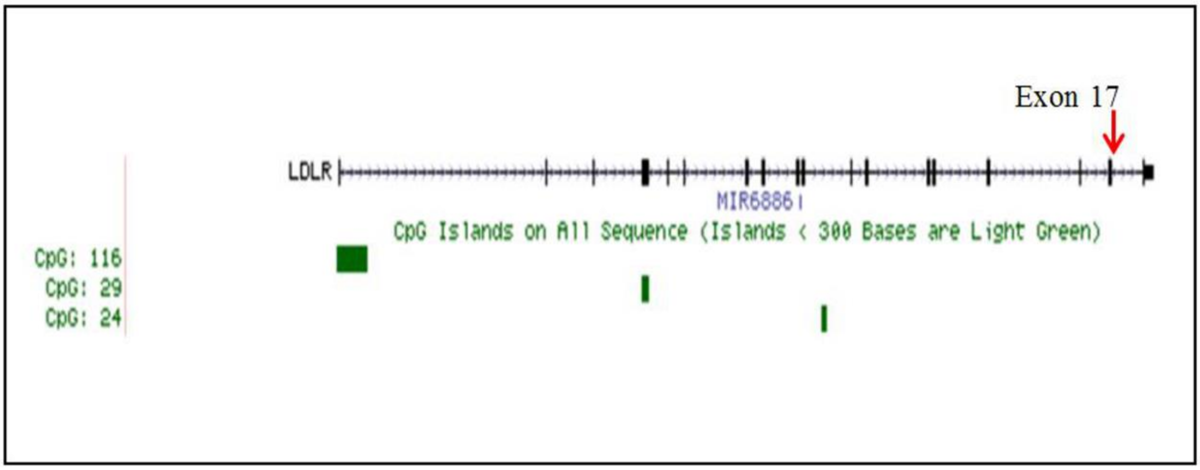

Figure S2: The CpG island map of *LDLR* gene by ENCODE, displayed using UCSC genome Browser

Green boxes are indicating CpG islands. CpG island map shows that *LDLR* gene has three CpG islands, in the promoter and 5'UTR (CpG: 116), in exon 4 (CpG: 29), and in intron 10 (CpG: 24).

Target	Orientation	sequences
LDLR promoter	Forward	CAGCTCTTCACCGGAGACCC
	Reverse	ACCTGCTGTGTCCTAGCTGG
LDLR- Exon 1	Forward	ACTCCTCCCCCTGCTAGAAACCTCA
	Reverse	CTATTCTGGCGCCTGGAGCAAGCC
LDLR- Exon 2	Forward	TTGAGAGACCCTTTCTCCTTTTCC
	Reverse	GCATATCATGCCCAAAGGGG
LDLR- Exon 3	Forward	TCAGTGGGTCTTTCCTTTGAG
	Reverse	CAGGACCCCGTAGAGACAAA
LDLR- Exon 4.1	Forward	TGGTGTTGGGAGACTTCACA
	Reverse	CACTCATCCGAGCCATCTTC
LDLR- Exon 4.2	Forward	AAGTGCATCTCTCGGCAGTT
	Reverse	CCCCTTGGAACACGTAAAGA
LDLR- Exon 4.3	Forward	AGCTTCCAGTGCAACAGCTC
	Reverse	CATACCGCAGTTTTCCTCGT
LDLR- Exon 4.4	Forward	TGTTCCAAGGGGACAGTAGC
	Reverse	AAATCACTGCATGTCCCACA
LDLR- Exon 5	Forward	AGAAAATCAACACACTCTGTCCTG
	Reverse	GGAAAACCAGATGGCCAGCG
LDLR- Exon 6	Forward	TCCTCCTTCCTCTCTCTGGC
	Reverse	TCTGCAAGCCGCCTGCACCG
LDLR- Exon 7	Forward	GGCGAAGGGATGGGTAGGGG
	Reverse	GTTGCCATGTCAGGAAGCGC
LDLR- Exon 8	Forward	CATTGGGGAAGAGCCTCCCC
	Reverse	GCCTGCAAGGGGTGAGGCCG
LDLR- Exon 9	Forward	TCCATCGACGGGTCCCCTCTGACCC
	Reverse	AGCCCTCATCTCACCTGCGGGCCAA
LDLR- Exon 10.1	Forward	AGATGAGGGCTCCTGGTGCGATGCC
	Reverse	GCCCTTGGTATCCGCAACAGAGACA
LDLR- Exon 10.2	Forward	GATCCACAGCAACATCTACTGGACC
	Reverse	AGCCCTCAGCGTCGTGGATA
LDLR- Exon 11	Forward	TCCTCCCCCGCCCTCCAGCC
	Reverse	GCTGGGACGGCTGTCCTGCG
LDLR- Exon 12	Forward	GCACGTGACCTCTCCTTATCCACTT
	Reverse	CACCTAAGTGCTTCGATCTCGTACG
LDLR- Exon 13	Forward	GTCATCTTCCTTGCTGCCTG
	Reverse	GTTTCCACAAGGAGGTTTCAAGGTT
LDLR- Exon 14	Forward	GAATCTTCTGGTATAGCTGAT
	Reverse	GCAGAGAGAGGCTCAGGAGG
LDLR- Exon 15	Forward	GAAGGGCCTGCAGGCACGTGGCACT
	Reverse	GTGTGGTGGCGGGCCCAGTCTTT
LDLR- Exon 16	Forward	CCTTCCTTTAGACCTGGGCC
	Reverse	CATAGCGGGAGGCTGTGACC
LDLR- Exon 17	Forward	GGGTCTCTGGTCTCGGGCGC
	Reverse	GGCTCTGGCTTTCTAGAGAGGG
LDLR- Exon 18	Forward	GCCTGTTTCCTGAGTGCTGG
	Reverse	TCTCAGGAAGGGTTCTGGGC

Table S1: Primers used to amplify DNA fragments of *LDLR*
Exon 4of the *LDLR* was screened by four overlapping PCR fragments (4.1, 4.2, 4.3, and 4.4) and exon 10 by two overlapping fragments (10.1, and 10.2).

ID	Gender	age	TC mmol/l	LDL mmol/l	LP(a) mg/dL	Drug therapy	Family history of hyperlipidaemia	City	TX	Clinical history of MI
FH1-P	M	19	11.4	10.0		Statin	Yes	Esfarayen	Yes	MI
FH1-F	M	50	3.1	4.8	92	-	Yes	Esfarayen	No	MI
FH1-M	F	45	7.1	4.7	96	-	Yes	Esfarayen	No	-
FH1-b	M	8	6.0	4.3	112	-	Yes	Esfarayen	No	-
FH2-P	F	1	18.6	14.2	60	Statin	Yes	Esfarayen	Yes	-
FH2-F	M	32	7.8	7.8	33	-	Yes	Esfarayen	No	-
FH2-M	F	30	6.5	3.5	54	-	Yes	Esfarayen	No	-
FH3-P	F	3	21.5	16.1	85	Statin	Yes	Mashhad	Yes	-
FH3-F	M	30	6.6	4.2	11	-	Yes	Mashhad	No	-
FH3-M	F	28	6.9	4.2	58	-	Yes	Mashhad	No	-
FH4-P	M	2	20.7	15.0	19	Ezetimibe	Yes	Neyshabor	Yes	-
FH4-F	M	42	5.6	3.4	142	-	Yes	Neyshabor	No	-
FH4-M	F	31	5.9	3.3	23	-	Yes	Neyshabor	No	-
FH4-S1	F	9	6.0	3.5	43	-	Yes	Neyshabor	No	-
FH4-S2	F	12	4.7	2.5	48	-	Yes	Neyshabor	No	-
FH5-P	M	11	15.5	7.8	112	Statin	Yes	Neyshabor	Yes	MI
FH5-F	M	37	8.1	5.6	29	-	Yes	Neyshabor	No	-
FH5-M	F	35	6.5	4.5	120	-	Yes	Neyshabor	No	-
FH5-b	M	1	2.6	1.4	<9	-	Yes	Neyshabor	No	-
FH6-P	F	6	22.6	16.6	24	Ezetimibe	Yes	Mashhad	Yes	-
FH6-M	F	40	9.8	7.1	24	_	Yes	Mashhad	No	-
FH7-P	M	3	22.5	17.5	41	Statin	Yes	Ghuchan	Yes	-
FH7-M	F	19	13.5	7.8	51	-	Yes	Ghuchan	No	-
FH8-P	M	10	21.7	19.4	-	Statin, Fish oil	Yes	Bandar Abbas	Yes	MI
FH8-F	M	40	7.8	5.2	-	Statin	Yes	Bandar Abbas	No	-
FH8-M	F	39	9.3	5.7	-	Statin	Yes	Bandar Abbas	Yes	-
FH8-b	M	1	2.8	1.7	-	-	Yes	Bandar Abbas	No	-
FH9-P1	F	12	5.2	3.3	249.4	Ezetimibe	Yes	Shahrekord	No	MI
FH9-P2	M	9	5.0	3.2	233.4	Ezetimibe	Yes	Shahrekord	No	-
FH9-F	M	39	5.2	3.6	8.3	Statin	Yes	Shahrekord	No	-
FH9-M	F	36	5.7	3.9	216.3	-	Yes	Shahrekord	No	-
FH10-P1	F	9	5.1	3.1	73.3	Statin,	Yes	Shahrekord	No	-
FH10-P2	M	7	6.5	4.9	-	Ezetimibe, Fish oil	Yes	Shahrekord	No	-
FH10-F	M	37	4.2	4.0	-	Statin	Yes	Shahrekord	No	-
FH10-M	F	34	5.6	3.5	119.7	-	Yes	Shahrekord	No	-
FH11-P	M	5	16.6	14.9	<9	Statin	Yes	Gorgan	Yes	-
FH11-F	M	30	5.2	10	55	-	Yes	Gorgan	No	-
FH11-M	F	24	13.9	12.9	<9	-	Yes	Gorgan	No	-
FH12-P	M	8	14.8	12.2	25	Statin	Yes	Gorgan	Yes	-

FH12-F	M	31	11.1	9.0	68	-	Yes	Gorgan	No	-
FH12-M	F	26	5.3	6.1	27	-	Yes	Gorgan	No	-
FH13-P	M	9	16.8	14.2	226	Statin, Ezetimibe	Yes	Gorgan	Yes	-
FH13-F	M	37	5.1	4.2	20	-	Yes	Gorgan	No	-
FH13-M	F	34	7.0	5.2	81	-	Yes	Gorgan	No	-
FH13-b1	M	4	6.8	4.7	74	-	Yes	Gorgan	No	-
FH13-b2	M	11	5.5	3.7	130	-	Yes	Gorgan	No	-
FH14-P	M	10	16.5	15.0		Statin, Ezetimibe	Yes	Ahwaz	Yes	MI
FH14-F	M	41	9.8	6.6	-	-	Yes	Ahwaz	No	-
FH14-M	F	35	8.5	6.8	-	-	Yes	Ahwaz	No	-
FH14-b	M	10	5.7	2.6	-	-	Yes	Ahwaz	No	-
FH15-P	M	9	18.6	13	-	Statin, Ezetimibe	Yes	Ahwaz	Yes	-
FH15-F	M	39	7.6	5.0	-	Statin	Yes	Ahwaz	No	-
FH15-M	F	38	7.9	6.1	-	Statin	Yes	Ahwaz	No	-
FH16-P	M	2	5.5	3.8	-	Statin, Ezetimibe	Yes	Ahwaz	Yes	-
FH16-F	M	31	7.0	6	-	-	Yes	Ahwaz	No	-
FH16-M	F	27	9.3	7.2	-	-	Yes	Ahwaz	No	-
FH17-P	F	4	15.0	13.6	-	Statin, Ezetimibe	Yes	Ahwaz	Yes	-
FH17-F	M	33	6.7	4.2	-	-	Yes	Ahwaz	No	-
FH17-M	F	28	5.9	4.3	-	-	Yes	Ahwaz	No	-

Table S2: Clinical and lipid parameters

For subjects who are under treatment, the lipid levels shows in the table were measured after treatment, as pre-treatment levels were not available. The two subjects included in the genetic screen who did not conform to the clinical diagnosis of HoFH are shown in bold.
